# Antitumor Potential of Extracellular Vesicles Released by Genetically Modified Murine Colon Carcinoma Cells With Overexpression of Interleukin-12 and shRNA for TGF-β1

**DOI:** 10.3389/fimmu.2019.00211

**Published:** 2019-02-13

**Authors:** Joanna Rossowska, Natalia Anger, Katarzyna Wegierek, Agnieszka Szczygieł, Jagoda Mierzejewska, Magdalena Milczarek, Bożena Szermer-Olearnik, Elżbieta Pajtasz-Piasecka

**Affiliations:** Ludwik Hirszfeld Institute of Immunology and Experimental Therapy, Polish Academy of Sciences, Wroclaw, Poland

**Keywords:** tumor-derived exosomes, tumor-derived microvesicles, dendritic cells, interleukin 12, TGF-β1 silencing, lentivectors, colon carcinoma, immunotherapy

## Abstract

Recent developments demonstrate that tumor-derived extracellular vesicles (EVs) could become a highly effective tool for delivery of antitumor factors. The main objective of the study was to determine whether EVs secreted by MC38 colon carcinoma cells genetically engineered for overproduction of interleukin (IL-)12 and/or shRNA targeting TGF-β1 are effectively loaded with these molecules and whether the obtained EVs could be an efficient tool for antitumor therapy. Fractions of EVs released by genetically modified MC38 cells [both modified tumor-derived exosomes (mTEx) and modified microvesicles (mTMv)] and those released by unmodified, wild-type MC38 cells were characterized in terms of loading efficacy, using real-time PCR and ELISA, as well as their antitumor potential. In order to examine the therapeutic potential of mTEx, they were applied in the form of sole treatment as well as in combination with dendritic cell (DC)-based vaccines stimulated with mTMv in the therapy of mice with subcutaneously growing MC38 tumors. The results demonstrated that genetic modification of wild-type MC38 tumor cells is an effective method of loading the molecules of interest into extracellular vesicles secreted by the cells (both TEx and TMv). The results also showed that mTEx secreted by cells engineered for overproduction of IL-12 and/or shRNA for TGF-β1 are able to induce tumor growth inhibition as opposed to TEx from unmodified MC38 cells. Additionally, antitumor therapy composed of mTEx (especially those deprived of TGF-β1) and DC-based vaccines allowed for regeneration of antitumor immunity and induction of the systemic Th1 response responsible for the sustained effect of the therapy. In conclusion, tumor-derived exosomes loaded with IL-12 and/or deprived of TGF-β1 could become an efficient adjuvant supporting induction of a specific antitumor response in both immuno- and chemotherapeutic schemes of treatment.

## Introduction

Tumor development is dependent on reliable communication and interaction between tumor cells and other cellular components of the tumor microenvironment (TME) (1). Recent scientific reports indicate that both tumor and tumor-infiltrating cells secrete large amounts of extracellular vesicles (EVs), which were proved to be an important element of intercellular communication within and beyond the tumor (2, 3). Scientific literature distinguishes at least two major classes of EVs: exosomes and microvesicles. Exosomes, which have an approximate size of 30–200 nm, are formed from the membrane of late endosomes [multivesicular bodies (MVBs)] by its inward budding. They are released from the endosome to the extracellular space in the process of MVB fusion with the plasma membrane. By contrast, microvesicles (200–1,000 nm) are formed through direct budding of the plasma membrane (1, 4). Both of them are potent vectors capable of transporting biologically active molecules (proteins, lipids, RNA, DNA) to target cells. Depending on the final cargo they can induce a wide range of processes that support cancer development, including immune suppression, cell proliferation, angiogenesis, epithelial-to-mesenchymal transition (EMT) and metastasis (5–7). However, given that tumor-derived exosomes (TEx) as well as microvesicles (TMv) are an easily accessible source of tumor antigens, express tumor-specific integrins which direct their migration toward the tumor site or predicted metastatic sites (8), and act as efficient carriers of different biological structures, they seem to be ideal vehicles for delivery of a broad range of therapeutic agents including non-coding RNAs, mRNAs, proteins, and synthetic drugs. The passive loading through the physical mixing of EVs with drugs (i.e., paclitaxel, cisplatin, curcumin), as well as active loading of molecules such as small interfering RNA by electroporation or sonication, are the most common methods to obtain EVs with desired cargo (9–12). EVs with modified content can also be obtained from genetically engineered cells with overexpression of desired molecules (13–15).

Disorders of immune cells caused by highly immunosuppressive TME are the most common reasons for poor results of cancer immunotherapy (16–18). Hence, therapeutic strategies allowing for efficient reprogramming of the hostile TME abundant in suppressive cytokines such as TGF-β, IL-10, or VEGF are intensively studied (19–21). TME can be modulated both by delivery of inflammatory cytokines, i.e., IL-12 or IL-2 and by elimination of suppressing factors. In our work we focused attention on TGF-β1 and IL-12, which play opposite functions in a tumor development. TGF-β1 is an anti-inflammatory cytokine associated with tumor progression and metastasis, often correlated with poor prognosis in patients (22). By contrast, IL-12 is a proinflammatory cytokine with high antitumor potential but also with high toxicity when applied in the recombinant form (23). The main purpose of our study was to develop and analyze TGF-β1-deprived EVs with IL-12 cargo. The TEx and TMv with modified content, referred to here generally as mTEx and mTMv, were obtained from genetically engineered murine colon carcinoma MC38 cells with overexpression of IL-12 and/or shRNA for TGF-β1 (MC38/IL12, MC38/shTGFβ1, or MC38/IL12shTGFβ1). The particles released by unmodified, wild-type MC38 cells, referred to here as TEx MC38 or TMv MC38, were applied as a control. Since EVs are released by cells in response to stress conditions such as hypoxia, acidosis, or oxidation stress (24), we decided to induce EVs secretion by culture of MC38 cells in hypoxic conditions. TEx and TMv fractions were isolated from the wild-type or genetically modified MC38 culture supernatant and separated on the basis of size using sequential centrifugation. The gathered data indicate that genetic modification of tumor cells is an efficient method to change the tumor-derived EVs' cargo to a therapeutic one. Moreover, immunotherapy composed of mTEx, especially those obtained from MC38/shTGFβ1 or MC38/IL12shTGFβ1, and dendritic cells (DCs) stimulated with mTMv from MC38/shTGFβ1 or MC38/IL12shTGFβ1 cells, applied in the treatment of mice with a subcutaneously growing MC38 tumor, induced tumor growth inhibition accompanied by a reduced number of MDSCs in the tumor and enhanced local and systemic Th1-type antitumor activity.

## Materials and Methods

### Mice

Female C57BL/6 mice were obtained from the Center for Experimental Medicine of the Medical University of Bialystok (Bialystok, Poland). All experiments were performed in accordance with EU Directive 2010/63/EU for animal experiments and were approved by the 1st Local Ethics Committee for Experiments with the Use of Laboratory Animals, Wroclaw, Poland (authorization number 33/2018). After the experiments, the mice were sacrificed by cervical dislocation.

### Cell Culture

The *in vivo* growing cell line of MC38 murine colon carcinoma from the Tumor Bank of the TNO Radiobiology Institute, Rijswijk, Holland, was adapted to *in vitro* conditions as described by Pajtasz-Piasecka et al. (25). The cell culture was maintained in RPMI 1640 (Gibco) supplemented with 100 U/ml penicillin (Polfa), 100 mg/ml streptomycin (Polfa), 1 mM sodium pyruvate (Sigma-Aldrich), 2-mercaptoethanol (Sigma-Aldrich) here called complete medium (CM), and 5% fetal bovine serum (FBS, Sigma-Aldrich). The genetically modified, stable MC38 cell lines with overexpression of murine IL-12 (MC38/IL12) and/or shRNA targeting mRNA for TGF-β1 (MC38/IL12shTGFβ1, MC38/shTGFβ1) were obtained after transduction of the wild-type MC38 cell line with lentiviral vectors encoding murine interleukin 12 (*mil12*) genes (VectorBuilder) or shRNA for TGF-β1 (EzBiolab). Transduced MC38/IL12 or MC38/shTGFβ1 cells were maintained in standard culture medium for MC38 cells supplemented with Geneticin 418 (Gibco, 1 mg/ml) or puromycin (Gibco, 10 μg/ml), respectively. The double transduced MC38/IL12shTGFβ1 cells were selected using two antibiotics simultaneously. The efficacy of overexpression of IL-12 and silencing of TGF-β1 in MC38 cells cultured in normoxic or hypoxic conditions was estimated by real-time PCR. Total RNA was isolated using a NucleoSpin RNA kit (Macherey-Nagel) and reverse-transcribed with a RevertAid First Strand cDNA Synthesis Kit (Thermo Fisher). Real-time PCR was performed using TaqMan Universal PCR Master Mix and TaqMan Gene Expression Assay primers for IL-12 and TGF-β1 (Applied Biosystems) in reference to the HPRT gene expression. Production of IL-12 or TGF-β1 was measured by ELISA (eBioscience) in supernatant harvested from MC38 cells cultured for 24 or 48 h (0.5 × 10^6^ cells/ml). DCs were differentiated from bone marrow of C57BL/6 mice according to the protocol described in our previous publication (26). The cells were cultured in CM supplemented with 10% FBS in the presence of rmGM-CSF (ImmunoTools, 40 ng/ml) and rmIL-4 (ImmunoTools, 10 ng/ml). After 6 days the loosely attached immature dendritic cells were stimulated with exosomes or microvesicles isolated from wild-type or transduced MC38 cell lines and used for further tests.

### Isolation and Characteristics of MC38 Tumor-Derived Particles

The production of TEx and TMv by MC38 cells was induced by culture of the cells in hypoxic conditions. Wild-type and genetically modified MC38 cells were seeded on multilayer flasks (Merck-Millipore) at the final density of 250 × 10^3^ cells/ml and cultured in the CM supplemented with 5% exosome-deprived FBS (Sigma-Aldrich) in hypoxic conditions (1% O_2_) for 48 h. Then the culture supernatants were harvested. TEx and TMv were isolated from the supernatants according to the procedure described by Felicetti (27) using sequential centrifugation at 2,000, 10,000 and 100,000 × *g* (Figure 2A). The TMv fraction was collected after centrifugation at 10 000 × g, while TEx fraction was collected after ultracentrifugation. Both fractions were then washed in PBS (IIET) filtered through 0.2 μm filters (Merck Millipore). To determine the number of TEx and TMv in the final suspension we used the flow cytometry method under the control of Absolute Counting Beads (Thermo Fisher) and 1 μm beads (Polysciences INC). After isolation particles were re-suspended in PBS (IIET) filtered through 0.2 μm filters (Merck Millipore). During the analysis the TEx and TMv were separated from flow cytometer- and PBS-derived debris using CFSE staining (Thermo Scientific, 2.5 μM). The quality of the obtained fractions of TEx and TMv was evaluated using transmission electron microscopy (TEM), dynamic light scattering (DLS), flow cytometry (FC), and western blotting (WB).

#### The Dynamic Light Scattering Method

The dynamic light scattering method was used for measurement of the particle size distribution and the purity of obtained fractions. Isolated particles were resuspended in filtered PBS and then the suspension was evaluated using a DLS Zetasizer (Malvern).

#### TEM

TEx and TMv fractions were fixed in 2% paraformaldehyde (Serva) and allowed to adsorb onto formvar carbon-coated grids for 20 min. The grids were then washed in PBS (IIET), fixed in 1% glutaraldehyde (Sigma-Aldrich) for 5 min and washed with water (7 × 2 min). Then each grid was transferred to a drop of uranyl-oxalate (4% uranyl acetate and 0.15 M oxalic acid in 1:1 v:v ratio; Sigma-Aldrich) at pH 7 for 5 min. At this stage, samples were counterstained using two protocols: 1. with uranyl acetate or 2. with methylcellulose. 1: The grid was transferred to a drop of 2% uranyl acetate (Chemapol) for 5 min and washed with a drop of water 3 times. Then the grids were allowed to air dry for 10 min. 2: The grids were then embedded in 2% methylcellulose (Sigma-Aldrich) with uranyl acetate (9:1 v:v ratio) for 10 min on ice. The excess of methylcellulose was removed from grids by filter paper and grids were allowed to air dry for 20 min. Preparations were visualized using a JEOL JEM-1200 EX 80 kV TEM.

#### Western Blotting

MC38 cells, TEx and TMv were washed twice in PBS (IIET) and lysed in RIPA buffer supplemented with protease inhibitor cocktail (both Sigma-Aldrich). Lysates were purified by centrifugation for 10 min at 4°C and 10,000 x *g* followed by supernatant transfer to new tubes. Total protein concentration in lysates was analyzed using the modified Lowry method (Bio-Rad) according to the manufacturer's protocol. Samples containing 20 μg (cell lysates), 100 μg (TEx lysates), or 10 μg (TMv lysates) of proteins were denatured in Laemmli Sample Buffer (Bio-Rad) supplemented with β-mercaptoethanol (Sigma-Aldrich) for 5 min at 100°C and then separated in 4–20% mini-protean TGX gels (Bio-Rad, USA). After the electrophoresis, proteins were transferred from gels to polyvinylidene difluoride (PVDF) membranes (0.45 μm; Merck Millipore). After blocking in 5% non-fat dry milk in 0.1% TBS/Tween-20 (TBST) at room temperature for 1 h, membranes were washed three times for 5 min in 0.1% TBST and then incubated overnight at 4°C with the following primary rabbit antibodies: monoclonal anti-CD81, monoclonal anti-TSG101, monoclonal anti-calnexin, monoclonal anti-CD9 (all from Abcam) or polyclonal anti-GM130 (Proteintech). After incubation with a primary antibody, the membrane was washed four times for 5 min in 0.1% TBST and then incubated at room temperature for 1 h with horseradish peroxidase conjugated secondary anti-rabbit antibody (DAKO). After incubation, the membranes were washed four times for 5 min in 0.1% TBST and protein bands were detected using luminol-based enhanced chemiluminescent substrate according to the manufacturer's protocol (Thermo Scientific). Images were acquired with G:BOX iChemi XR (Syngene).

#### Flow Cytometry

The flow cytometry method was applied to determine the expression of CD63, CD9, and CD81 on the surface of isolated particles. Isolated TEx or TMv were resuspended in PBS filtered through 0.2 μm filters (Merck Millipore) and then labeled with monoclonal antibodies conjugated with fluorochromes: anti-CD63 APC, anti-CD9 APC, anti-CD81 APC, rat IgG2aκ APC and Armenian hamster IgG APC isotype controls (all from BioLegend). The expression of cell surface markers was analyzed using FACS Fortessa with FACSDiva software (Becton Dickinson).

#### Determination of TGF-β1 and IL-12 Levels in Isolated Particles

Levels of mRNA for IL-12 and TGF-β1 in particles isolated from MC38 cells were measured by real-time PCR. Total RNA was isolated using the NucleoSpin RNA XS kit (Macherey-Nagel) and reverse-transcribed with the SuperScript III First-Strand Synthesis System (Thermo Fisher). Real-time PCR was performed using TaqMan Universal PCR Master Mix and TaqMan Gene Expression Assay primers for IL-12 and TGF-β1 (Applied Biosystems) in reference to the HPRT gene expression. The IL-12 and TGF-β1 proteins inside the TEx and TMv were evaluated by measurement of the cytokine concentration by ELISA (eBioscience) in lysate prepared from isolated particles using Lysis Buffer for ELISA kits (RayBiotech).

### Characteristics of Dendritic Cells Stimulated With MC38-Derived Particles

Immature DCs were stimulated with TEx and TMv obtained from wild-type or genetically modified MC38 tumor cells (5 × 10^6^ particles/1 × 10^6^ cells) in the presence of GM-CSF (40 ng/ml) for 24 h. After stimulation dendritic cells were harvested and labeled with monoclonal antibodies conjugated with fluorochromes: anti-CD40 PE (BD Biosciences), anti-CD80 PerCP-Cy5.5, anti-CD86 PE-Cy7, anti-MHC II FITC, anti-PD-L1 APC, and anti-CD11c BV650 (all from BioLegend). The expression of cell surface markers was analyzed using FACS Fortessa with FACSDiva software (Becton Dickinson). Moreover, the ability of TEx or TMv-stimulated DCs to activate naïve lymphocytes was evaluated. Stimulated DCs were co-cultured with splenocytes obtained from healthy mice in the final ratio of 1:10 for 5 days in CM supplemented with 10% FBS and 200 U/ml of recombinant human IL-2. After 5 days, the cells and supernatants were collected. Cytotoxic activity of primary stimulated splc toward DiO-labeled MC38 target cells, as well as the ability of the effector cells to secrete lytic granules, was measured as previously described by Rossowska et al. (28). The cytotoxic effector cells were identified by flow cytometry (FACS Fortessa) using the following monoclonal antibodies: anti-CD49b PE-CF594 (BD Biosciences), anti-CD8 PE-Cy7, and anti-CD107a APC (both from BioLegend). Production of IFN-γ by primed spleen cells was evaluated using commercially available ELISA kits (eBioscience) according to the manufacturer's instructions. A direct effect of EVs on splenocyte activity was evaluated as a concentration of IFN-γ in supernatants from 5-day culture of splenocytes in the presence of TEx or TMv.

### Therapeutic Treatment Schedule

Eight- to ten-week old female C57BL/6 mice were subcutaneously inoculated in the right flank with MC38 cells (1.1 × 10^6^/0.2 ml/mouse). The mice were treated according to the scheme presented in Figure 4A. Tumor-derived exosomes obtained from the wild-type MC38 cell line (TEx MC38) or genetically modified MC38/IL12 (TEx MC38/IL12), MC38/shTGFβ1 (TEx MC38/shTGFβ1), or MC38/IL12shTGFβ1 (TEx MC38/IL12shTGFβ1) cell lines were inoculated peritumorally (p.t.), twice per week for three consecutive weeks in a dose of 2 × 10^6^ particles/100 μl NaCl/mouse. On the 16th, 23rd, and 30th days, dendritic cell-based vaccines stimulated with wild-type or genetically modified MC38-derived TMv (DC/TMv MC38, DC/TMv MC38/IL12, DC/TMv MC38/shTGFβ1, or DC/TMv MC38/IL12shTGFβ1) were applied p.t. in the final number of 0.7 × 10^6^/0.2 ml/mouse. On the 35th day, mice were sacrificed, and their spleens and tumor nodules were dissected, homogenized and stored in liquid nitrogen for further analyses. The procedure of tumor growth monitoring was presented by Rossowska et al. (29). The therapeutic effect of the treatment was evaluated using tumor growth inhibition (TGI). Statistically significant differences were calculated using Friedman and Dunn's multiple comparison tests.

### Analysis of MC38 Tumor-Infiltrating Immune Cells

Tumor cells isolated from mice were thawed and stained for identification of myeloid or lymphoid cell subpopulations according to the procedure described by Rossowska et al. (30). Briefly, tumor-derived cells were stained with LIVE/DEAD Fixable Violet Dead Staining Kit (Thermo Fisher) and then stained with cocktails of fluorochrome-conjugated monoclonal antibodies: anti-CD3 PE-CF594, anti-CD19 PE-CF594, anti-CD49b PE-CF594 (all from BD Biosciences), anti-CD45 BV605, anti-CD11b PerCP-Cy5.5, anti-CD11c BV650, anti-F4/80 AlexaFluor 700, anti-Ly6C PE, anti-Ly6G APC-Cy7, anti-MHC II FITC, anti-CD86 PE-Cy7 (all from BioLegend) for myeloid cell identification, and anti-CD45 BV605, anti-CD3 BV650, anti-CD4 FITC, anti-CD8 APC/Fire 750, anti-CD25 PE, anti-CD44 PE-Cy7, anti-CD62L PerCP-Cy5.5 (all from BioLegend) for lymphocytes identification. Then, the cells were fixed using the FoxP3 Fixation Permeabilization Staining Kit (eBioscience). Tumor cells stained with myeloid or lymphocyte cocktail were additionally incubated with anti-CD206 APC (BioLegend) or anti-FoxP3 APC (eBioscience) antibodies, respectively. The analysis was performed using a FACS Fortessa flow cytometer with Diva software (Becton Dickinson).

### Evaluation of Systemic Antitumor Response

In order to determine the polarization of the systemic immune response followed by applied treatment, Tbet and FoxP3 expression, and IFN-γ production by T cells was measured. Spleen cells, obtained from treated and control mice, were stimulated with ConA (0.5 μg/ml; Sigma-Aldrich) and IL-2 (200 U/ml) for 48 h. Then cells were harvested and after staining with the fluorochrome-conjugated antibodies anti-CD4 FITC and anti-CD8 APC/Fire 750 (BioLegend) were fixed and permeabilized for intracellular staining of Tbet, FoxP3 and IFN-γ with the following antibodies: anti-Tbet PE-Cy7, anti-FoxP3 APC, anti-IFN-γ PE (eBioscience). Flow cytometry analyses were performed using FACS Fortessa with FACSDiva software (Becton Dickinson).

### Statistics

All the data were analyzed using GraphPad Prism 6 software. The cytometric data presentations were prepared using NovoExpress software. The statistical significance in kinetics of tumor growth was calculated using the Friedman test followed by Dunn's multiple comparison *post-hoc* test. In all remaining analyses the statistical differences were calculated using the nonparametric Kruskal-Wallis test for multiple independent groups followed by Dunn's multiple comparison *post-hoc* test. Differences with a *p*-value <0.05 were regarded as significant.

## Results

### Isolation and Characteristics of TEx and TMv Released by Genetically Modified MC38 Cells

**Figure 1:**
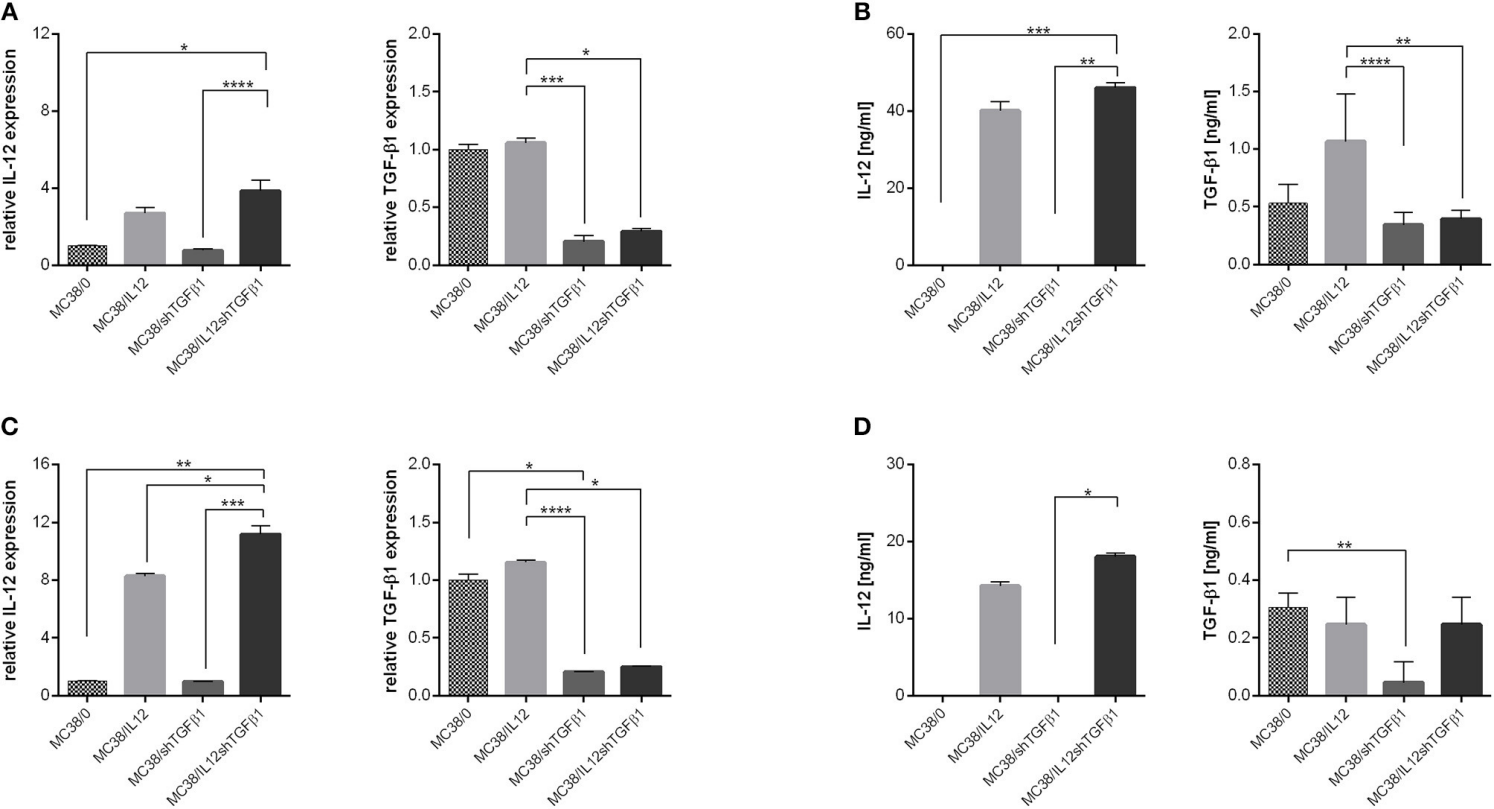
Effectiveness of IL-12 overexpression and TGF-β1 silencing in genetically modified MC38 cell lines. **(A,C)** Relative expression of IL-12 and TGF-β1 measured by real-time PCR in MC38 cells cultured in normoxic **(A)** or hypoxic **(C)** conditions. **(B,D)** Concentration of IL-12 and TGF-β1 in supernatants from MC38 cells cultured in normoxic **(B)** or hypoxic **(D)** conditions. The results are given as the mean ± SD calculated for at least two repeats in two independent experiments. The differences between the groups were estimated using the nonparametric Kruskal-Wallis test followed by Dunn's multiple comparison test (*p < 0.05, **p < 0.01, ***p < 0.001, ****p < 0.0001).

**Figure 2:**
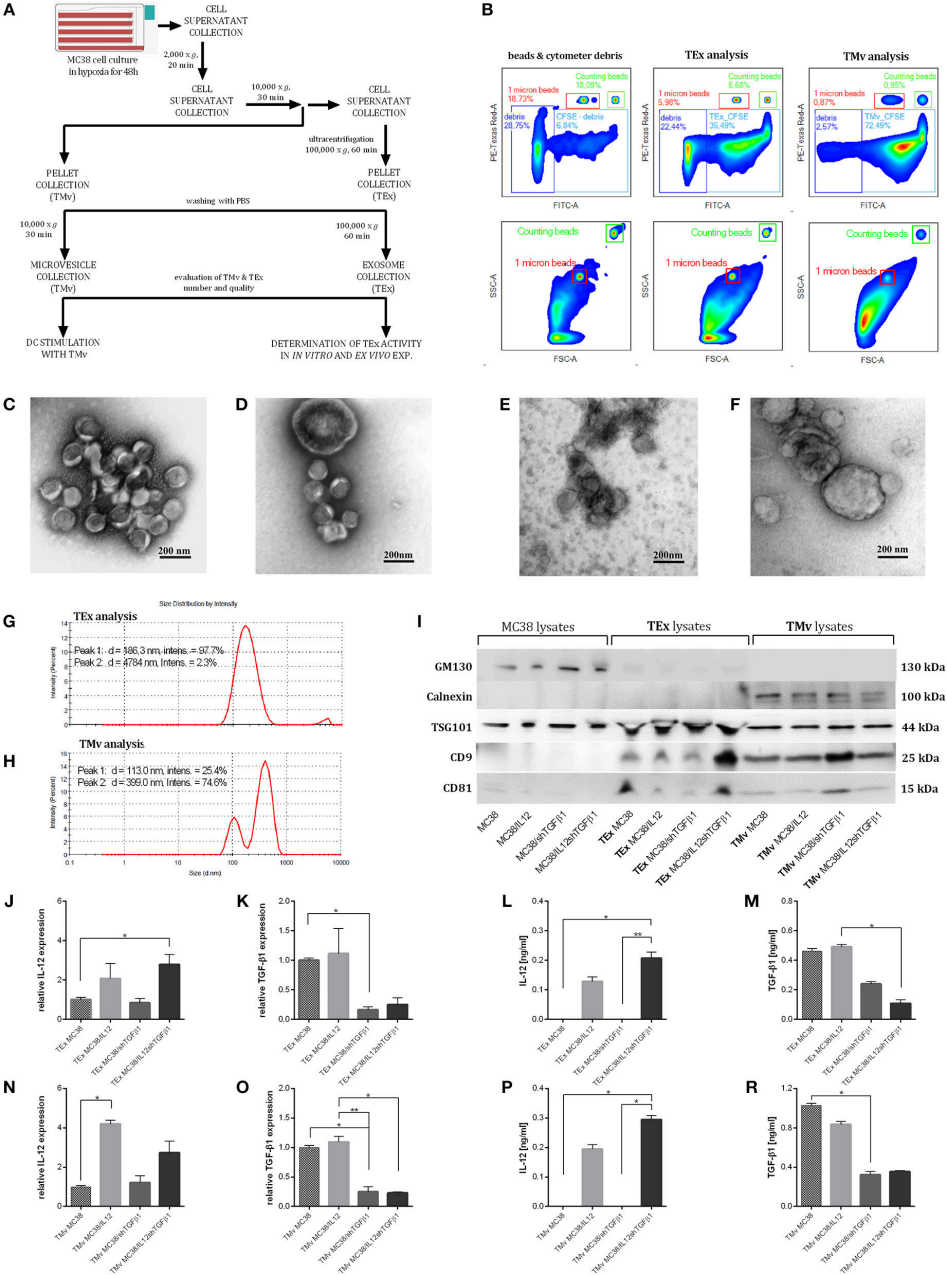
The method of isolation and characterization of TEx and TMv released by wild-type or genetically modified MC38. **(A)** Scheme of TEx and TMv isolation. **(B)** Representative density plots showing the method of evaluation and counting of CFSE stained TEx and TMv using the LSR Fortessa flow cytometer. The data are presented for the example of particles isolated from unmodified MC38 cells. TEM analysis of TEx **(C,E)** and TMv **(D,F)** counterstained with uranyl acetate **(C,D)** or with methylcellulose **(E,F)**. Magnification 100,000x. **(G,H)** Representative histograms showing the measurement of MC38-derived TEx and TMv particle size distribution using the DLS Zetasizer (Malvern). **(I)** WB analysis of CD81, CD9, TSG101, GM130, and calnexin in lysates from MC38 cell lines, TEx and TMv fractions. **(J,K,N,O)** Relative expression of mRNA for IL-12 or TGF-β1 in TEx and TMv isolated from wild-type or genetically modified MC38 cell lines. **(L,M,P,Q)** Concentration of IL-12 and TGF-β1 in lysates prepared from TEx and TMv isolated from wild-type or genetically modified MC38 cell lines measured using the ELISA. The results are given as the mean ± SD calculated for at least two repeats in two independent experiments. The differences between the groups were estimated using the nonparametric Kruskal-Wallis test followed by Dunn's multiple comparison test (*p < 0.05, **p < 0.01).

Tumor-derived EVs with modified content (mTEx and mTMv) were obtained from the murine colon carcinoma MC38 cell line with silenced expression of murine TGF-β1 and/or with overexpression of murine IL-12. The MC38/IL12 and MC38/shTGFβ1 cell lines were established by transduction with lentiviral vectors encoding *il12* genes or shRNA for TGF-β1 followed by geneticin or puromycin antibiotic selection, respectively. The MC38/IL12shTGFβ1 cell line was obtained by double transduction followed by selection in the presence of both antibiotics. The quality of the transduction process was monitored using real-time PCR and ELISA (Figures 1A,B). In order to stimulate TEx and TMv production by genetically modified or wild-type MC38 cell lines, the cells were cultured in hypoxic conditions for 48 h. To confirm that genetically engineered cells retain their properties in hypoxia we also monitored the IL-12 and TGF-β1 expression after culture of the cells in hypoxic conditions for 48 h (Figures 1C,D). Isolation of TEx and TMv from the culture supernatant was carried out according to the scheme presented in Figure 2A. After the final washing in PBS the sample from each isolated fraction was collected for further qualitative and quantitative analyses. The quantitative analysis was performed using the flow cytometric method. For this purpose, the samples from TEx and TMv fractions were stained with CFSE dye, fluorescence of which depends on the esterase activity inside the cells or, as in this case, inside particles. This method makes it possible to distinguish the TEx and TMv from flow cytometer and PBS-derived debris during cytometric analysis. The analysis was performed in the presence of counting beads to count the total number of CFSE-labeled TEx or TMv in suspension and reference 1 μm beads to show the approximate size of isolated particles. Representative density plots showing the example of cytometric analysis of TEx and TMv isolated from wild-type MC38 cells are presented in Figure 2B. All presented plots represent the result of acquiring 1,000 count beads per sample. Thus, the relation between control plots showing beads and debris and plots showing TEx or TMv is actual. The differences in the number of EVs secreted by the wild-type and genetically modified MC38 cells are presented in Supplementary Figure 1. However, it should be stressed that the final number of TEx in preparation may depend on differences in the TEx release as well as differences between proliferation rate of particular cell lines. The quality and size of particles obtained after isolation were measured using TEM and the DLS Zetasizer. The visualization of the EVs with TEM was performed using two protocols of counterstaining: the first with uranyl acetate (Figures 2C,D) and second with methylcellulose (Figures 2E,F). Both of them showed that the TMv fractions are contaminated by TEx, while the TEx fractions seems to be pure. The TEM analysis showed that the approximate size of TEx is 100 nm, whereas the size of TMv is >200 nm. Histograms presented in Figures 2G,H show differences in a size distribution, measured by DLS, between the TMv fraction obtained after centrifugation at 10,000 × *g* for 30 min and the TEx fraction achieved after ultracentrifugation at 100,000 × *g* for 60 min. Using this method we confirmed that the TMv fraction (mean size: 399 nm) was partially contaminated with small TEx particles (mean size: 123 nm), while the TEx fraction was more homogeneous and its mean size was 186 nm. We noted that the size of particles differs when measured by TEM and DLS. The TEM analysis revealed that there are a lot of aggregates in TEx or TMv suspensions. Thus, it seems probable that DLS measurements, which show the mean size of particles, are overstated due to the presence of aggregates in the suspension. There were no significant differences in size distribution between fractions from wild-type and genetically modified cells, thus only representative histograms for TEx and TMv isolated from the wild-type MC38 cell line were presented. In the next step we evaluated the expression of specific markers for exosomes by western blotting and flow cytometry. The western blotting method was used for analysis of both proteins specific for exosomes (CD9, CD81, and TSG101) and cell organelle proteins that exosomes should not contain (GM130 and calnexin). The comparative analysis of lysates from MC38 cell lines, TEx and TMv fractions revealed that GM130 and calnexin were not visible in the TEx-derived lysates, while TSG101, CD81, and CD9 were enriched in the TEx and TMv fractions (Figure 2I). The results indicate that TEx fractions were not contaminated with TMv or cell debris. Moreover, expression of CD81 and CD9 in the TMv fractions confirmed our previous observations showing contamination of TMv with TEx. Unfortunately, the comparative analysis of the TEx marker expression between particular cell lines were not possible, because we were not able to determine the precise concentration of total protein in the TEx fractions. We observed that TEx fractions were considerably contaminated with FBS-derived proteins like albumin and the final protein level in a sample was very high, although the concentration of TEx-derived proteins was certainly much lower. For this reason, we decided to perform additional analysis of expression of TEx specific markers using flow cytometry. The analysis showed that the expression of CD63, CD9, and CD81 on the TEx is higher than in TMv. We also noted the difference in expression of these molecules between TEx isolated from genetically modified and wild-type MC38 cells (Supplementary Figure 2).

Further studies demonstrated the changes in the content of TEx and TMv following genetic modifications of the MC38 cell line. We observed increased expression of IL-12, measured as mRNA and protein level, in TEx and TMv isolated from cells transduced with lentivectors encoding murine *il12* genes (Figures 2J,L,N,P), as well as diminished expression of mRNA and protein for TGF-β1 both in TEx and TMv isolated from MC38 cells with silenced expression of TGF-β1 (Figures 2K,M,O,Q). The obtained results indicate that the content of particles produced by tumor cells may be effectively changed following genetic modification of wild-type tumor cells.

### The Influence of mTEx and mTMv on Bone Marrow-Derived Dendritic Cell Activity

**Figure 3:**
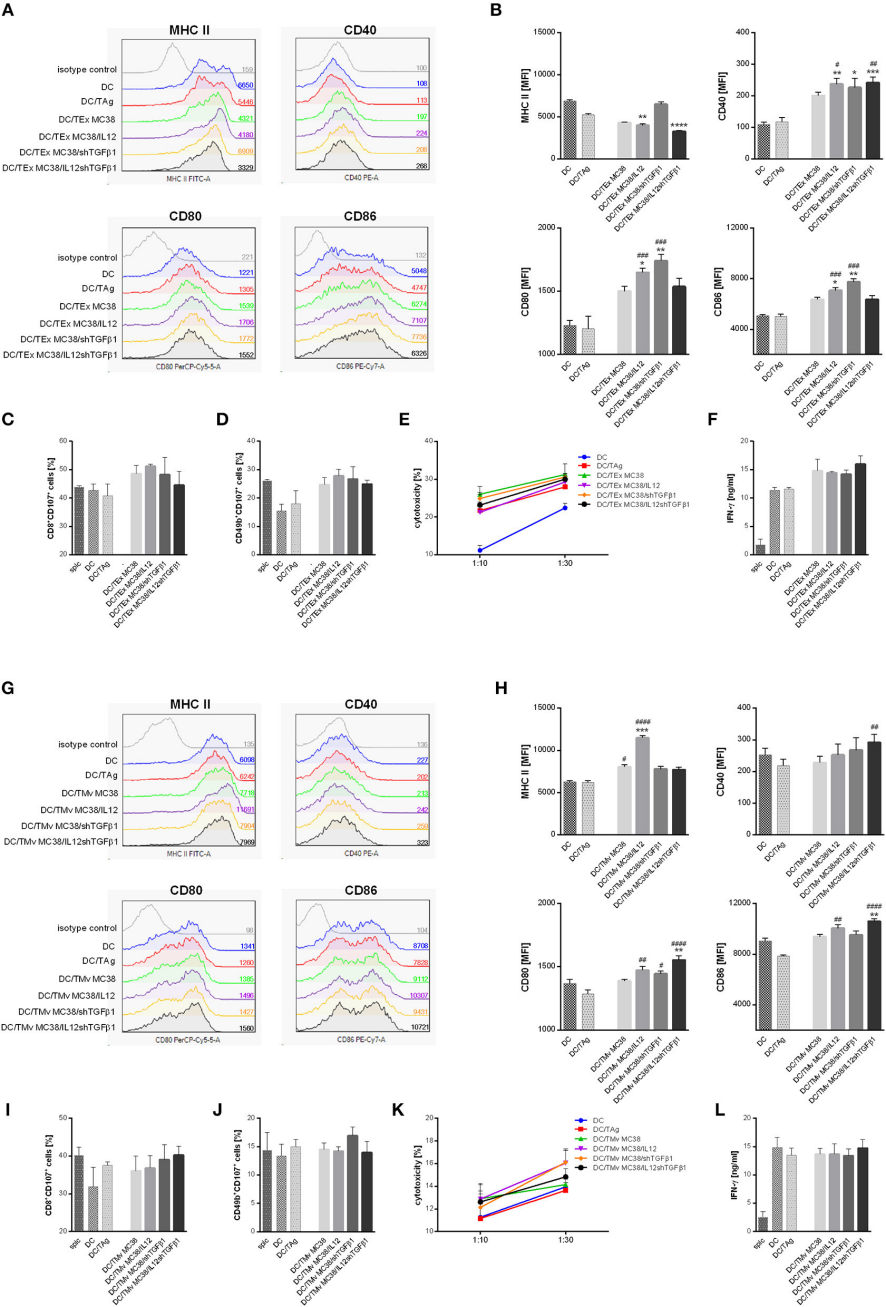
The influence of TEx or TMv isolated from wild-type MC38 cells or MC38 cells with overexpression of IL-12 and/or shTGFβ1 on dendritic cell (DC) activity *in vitro*. **(A,B)** Representative histograms and bar plots showing phenotypic changes of DCs after 24-h stimulation with TEx. **(C–F)** Splenocyte activity after primary stimulation with TEx-treated dendritic cells. The data show the percentage of CD8^+^CD107a^+^ CTLs **(C)** and CD49b^+^CD107a^+^ NK cells **(D)** among splenocytes obtained after 5-day co-culture with TEx-treated DCs, their cytotoxic activity toward MC38 tumor cells **(E)** and production of IFN-γ during co-culture of splc and DCs **(F)**. **(G,H)** Representative histograms and bar plots showing phenotypic changes of DCs after 24-h stimulation with TMv. **(I–L)** Splenocyte activity after primary stimulation with TMv-treated dendritic cells. The data show the percentage of CD8^+^CD107a^+^ CTLs **(I)** and CD49b^+^CD107a^+^ NK cells **(J)** among splenocytes obtained after 5-day co-culture with mTMv-treated DCs, their cytotoxic activity toward MC38 tumor cells **(K)** and production of IFN-γ during co-culture of splc and DCs **(L)**. The results are given as the mean ± SD calculated for three repeats in three independent experiments. The differences between the groups were estimated using the nonparametric Kruskal-Wallis test followed by Dunn's multiple comparison test (*p < 0.05, **p < 0.01, ***p < 0.001, ****p < 0.0001 in reference to DC; ^#^*p* < 0.05, ^*##*^*p* < 0.01, ^*###*^*p* < 0.001, ^*####*^*p* < 0.0001 in reference to DC/TAg).

After 24 h stimulation with mTEx or mTMv the changes in the phenotype of dendritic cells as well as their effectiveness in primary activation of naïve T cells isolated from spleen were evaluated. The results were related to the phenotype and activity of unstimulated DCs, DCs stimulated with lysate prepared by repeated freezing and thawing of wild-type MC38 cells (DC/TAg) or DCs stimulated with TEx or TMv isolated from wild-type MC38 cells (DC/TEx MC38 or DC/TMv MC38). The phenotype analysis showed that all types of TEx strongly affected the maturation of DCs (Figures 3A,B). Although after stimulation with TAg, TEx MC38 cells, and mTEx we observed a significant decrease in MHC II expression, the CD40, CD80, and CD86 co-stimulatory molecule expression on the DCs stimulated with TEx MC38 or mTEx considerably increased compared to DC or DC/TAg. The highest maturity stage was shown by DCs stimulated with TEx MC38/shTGFβ1 or with TEx MC38/IL12. Interestingly, TEx isolated from wild-type cells and TEx MC38/IL12shTGFβ1 induced similar phenotypical changes in DCs. Further analyses showed that DCs stimulated with TEx from unmodified MC38 cells or mTEx were more potent to activate cytotoxicity of T lymphocytes and NK cells toward wild-type MC38 cells than unstimulated DCs or DC/TAg (Figures 3C–E). Moreover, splenocytes produced more IFN-γ during co-culture with DC/TEx MC38 or DC/mTEx than those activated by DC/TAg (Figure 3F). Nevertheless, there were no significant differences between groups stimulated with TEx from unmodified MC38 cells and mTEx.

Dendritic cells stimulated with TMv from unmodified MC38 cells or mTMv also showed higher maturity than DC/TAg. All groups stimulated with microvesicles revealed higher expression of co-stimulatory and MHC class II molecules than control DCs and DC/TAg cells (Figures 3G,H). However, as in the case of stimulation with TEx, there were no significant changes between groups stimulated with TMv from wild-type MC38 cells or genetically modified MC38 cells. Although we observed similar antitumor activity of DC/TMv MC38 and DC/TAg, the DC/TMv MC38/IL12shTGFβ1 seems to be more effective in activation of cytotoxic CTLs (CD8^+^CD107a^+^), whereas DC/TMv MC38/shTGFβ1 was more potent in activation of cytotoxic NK cells (CD49b^+^CD107a^+^) than control DCs. Additionally, lymphocytes stimulated with these cells showed higher cytotoxic activity toward MC38 cells than in control groups (Figures 3I–K). In contrast to TEx, DC/TMv were not able to induce IFN-γ production at a higher level than control DCs and DC/TAg cells (Figure 3L). In summary, both mTEx and mTMv induced maturation of dendritic cells, but DCs stimulated with TEx, from both wild-type and genetically modified MC38 cells, seemed to be better activators of antitumor activity of lymphoid cells.

We also performed an experiment which aimed to determine a direct influence of TEx and TMv on splenocyte ability to produce IFN-γ. It was observed that splenocytes stimulated with mTEx or mTMv, especially these isolated from MC38 cells with overexpression of IL-12 produced IFN-γ on significantly higher level than unstimulated cells or stimulated with particles from wild-type MC38 cells (Supplementary Figure 3).

### Antitumor Activity of mTEx and DCs Stimulated With mTMv

**Figure 4:**
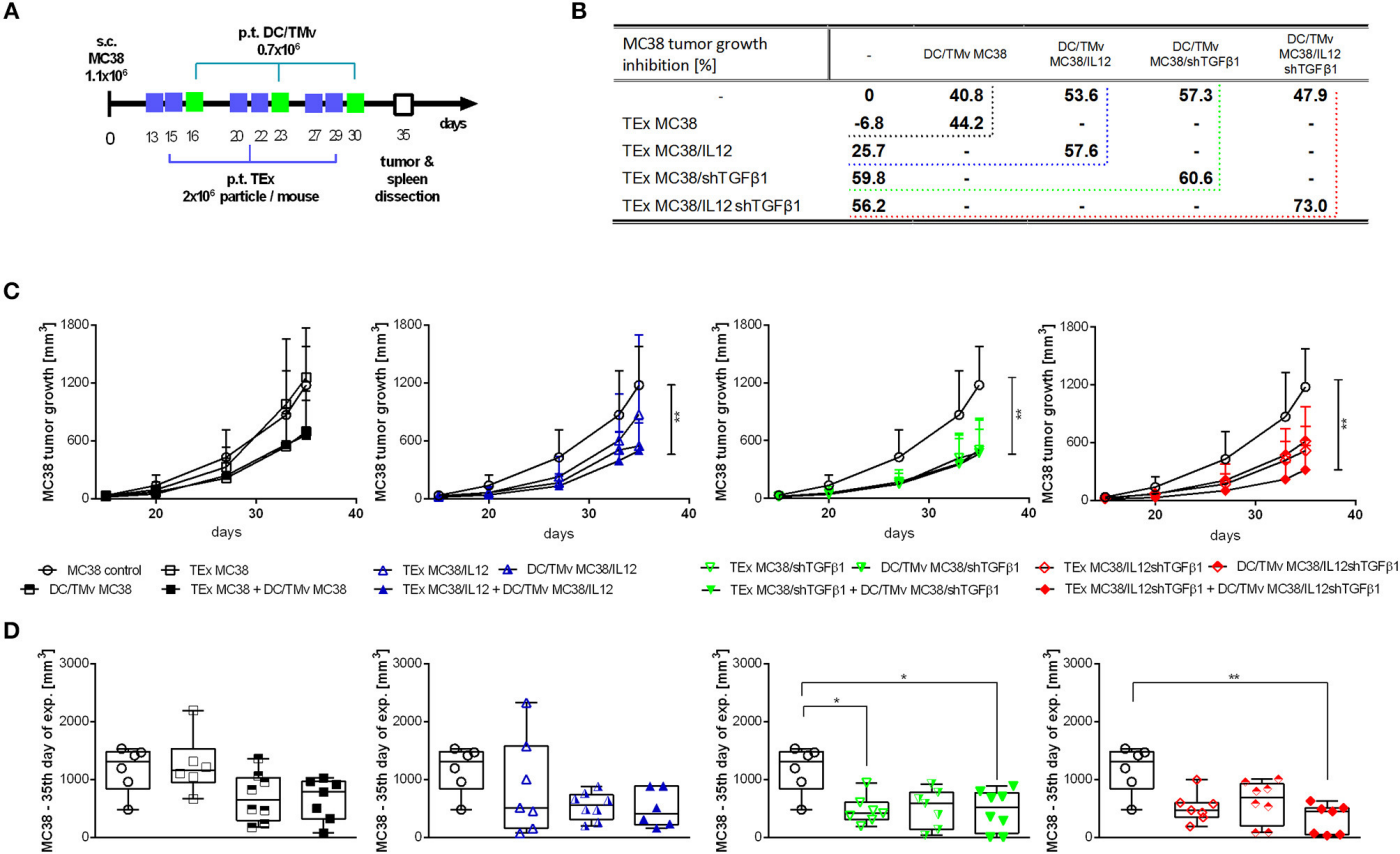
MC38 tumor growth after immunotherapy with mTEx and/or dendritic cells stimulated with mTMv. **(A)** Scheme of treatment. **(B)** Table presenting MC38 tumor growth inhibition (TGI) calculated on the 35th day of the therapeutic experiment in relation to the untreated group. **(C)** Curves presenting the mean tumor volume after immunotherapy. The differences between groups were estimated using the Friedman test (**p < 0.01). **(D)** The box graphs present the median tumor volume, calculated on the 35th day. To calculate the mean ± SD, 6–8 mice per group were analyzed. The differences between the groups were estimated using the nonparametric Kruskal-Wallis test followed by Dunn's multiple comparison test (*p < 0.05, **p < 0.01).

Based on the results obtained in *in vitro* assays, we decided to apply both mTEx and mTMv in the form of immunotherapy of mice with subcutaneously growing MC38 tumors. However, due to the higher activity, mTEx were applied in the form of a vaccine directly inoculated to the host, whereas mTMv were used mainly as the tumor antigen source to stimulate dendritic cell-based vaccines. The scheme of the combined treatment with TEx from unmodified MC38 cells or mTEx (TEx MC38/IL12, TEx MC38/shTGFβ1, TEx MC38/IL12shTGFβ1) and TMv from unmodified MC38 cells or mTMv (TMv MC38/IL12, TMv MC38/shTGFβ1, TMv MC38/IL12shTGFβ1)-stimulated DCs is presented in Figure 4A. The kinetics of MC38 tumor growth during therapy as well as the tumor growth inhibition calculated on the 35th day of therapy, are presented in Figures 4B–D. The obtained data showed that TEx MC38 accelerated tumor growth and in this case TGI (tumor growth inhibition calculated on the 35th day of the experiment in relation to the untreated MC38 control) reached −6.8%. For comparison, TEx MC38/IL12, TEx MC38/shTGFβ1 or TEx MC38/IL12shTGFβ1 induced TGI at the level of 25.7, 59.8, or 56.2%, respectively. Although TEx MC38/IL12shTGFβ1 did not induce the highest TGI, we observed that application of these exosomes in combination with DC/TMv MC38/IL12shTGFβ1 resulted in the best therapeutic effect and TGI reached 73%. In other groups, which received combinations of mTEx and DC/mTMv, the therapeutic effect did not differ significantly from that obtained for groups which obtained DC/TMv MC38/IL12 or DC/TMv MC38/shTGFβ1 alone. It should also be emphasized that the effect of application of DCs stimulated with mTMv was better than that observed for DCs stimulated with TMv from unmodified MC38 cells. However, the differences between TGI calculated for particular groups were not considerable (Figure 4B). Taking into account the particular genetic modifications of wild-type tumor cells, it seems that particles isolated from MC38 cells with silenced expression of TGF-β1 are the most important in induction of a potent therapeutic effect.

### The Influence of Therapy Composed of mTEx and DC/mTMv on the Local and Systemic Antitumor Immune Response

**Figure 5:**
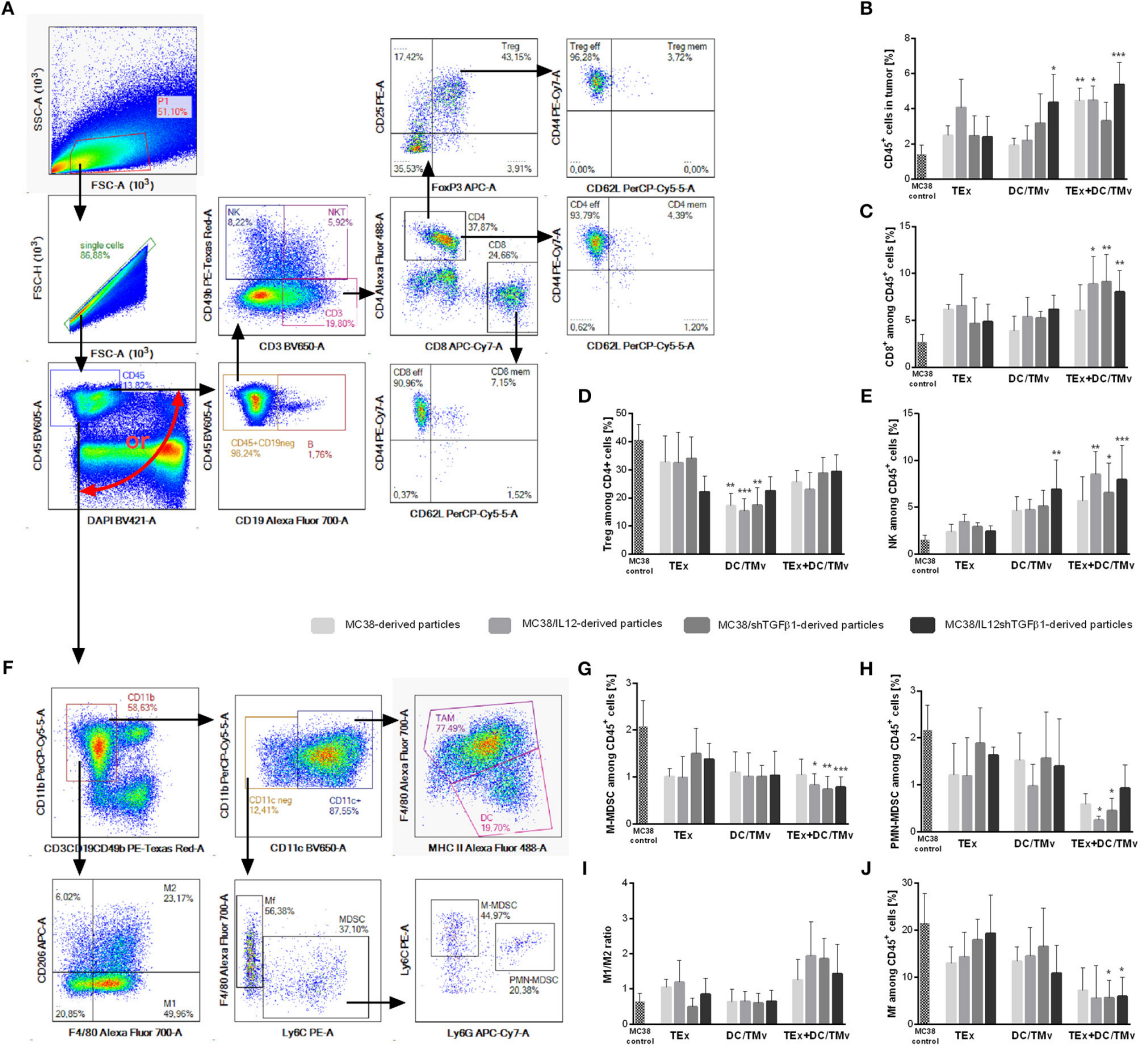
The influence of immunotherapy on the immune landscape in MC38 tumor nodules. **(A,F)** Schemes of multiparameter flow cytometric analyses showing the way of distinguishing lymphoid **(A)** or myeloid **(F)** cell subpopulations. **(B)** The percentage of leukocytes in tumor nodules. **(C–E,G,H,J)** Percentages of effector or suppressor cell subpopulations which underwent changes during therapy. **(I)** The M1/M2 ratio showing changes in polarization of tumor-infiltrating macrophages occurring during therapy. To calculate the mean ± SD, 6–8 mice per group were analyzed. The differences between the groups were estimated using the nonparametric Kruskal-Wallis test followed by Dunn's multiple comparison test (*p < 0.05, **p < 0.01, ***p < 0.001 in reference to MC38 control).

**Figure 6:**
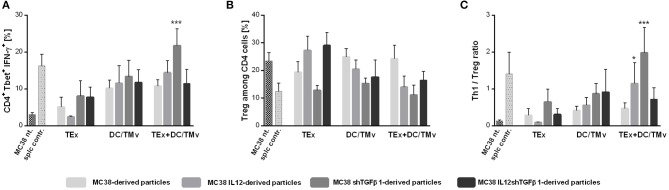
Polarization of immune response following mTEx and DC/mTMv treatment. **(A)** Bar plots presenting changes in Tbet expression and IFN-γ production in CD4^+^ cells from spleens of treated mice. **(B)** The percentage of Treg cells among CD4^+^ cells from spleens of treated mice. **(C)** The Th1/Treg ratio. To calculate the mean ± SD, 6–8 mice per group were analyzed. The differences between the groups were estimated using the nonparametric Kruskal-Wallis test followed by Dunn's multiple comparison test (*p < 0.05, ***p < 0.001 in reference to MC38 control).

We determined the influence of immunotherapy on changes in the immune composition of the MC38 tumor microenvironment applying multicolor flow-cytometric analyses. The lymphoid cell panel (Figure 5A) allowed for simultaneous identification of CTLs, Th, Treg, B, NK, and NKT cell subpopulations, as well as determination of their activity. Although all subpopulations were analyzed, we decided to present data for cells which underwent significant changes during applied therapy. The analysis of tumor nodules dissected on the 35th day of immunotherapy revealed very high influx of leukocytes (CD45^+^ cells; Figure 5B) after treatment with a combination of exosomes and DCs stimulated with microvesicles (both TMv and mTMv) as well as after DC/TMv MC38/IL12shTGFβ1. Excluding the group which received unmodified particles, the effect was accompanied by high infiltration of effector CTLs and NK cells (Figures 5C,E). We also observed a reduced population of Treg cells after combined therapy. However, a statistically lower percentage of the cells compared to the untreated control was noted only after application of monotherapy with TMv-stimulated DCs, especially after DC/TMv MC38/IL12 (Figure 5D). The changes occuring in the lymphoid populations were accompanied by modifications in the percentage of myeloid cells infiltrating tumor nodules. The applied myeloid cell panel (Figure 5F) allowed for simultaneous identification of TAM, DCs, resident macrophages (Mf), M-MDSCs and PMN-MDSCs as well as identification of the macrophage polarization stage through the evaluation of CD206 expression. We noted that percentages of M-MDSCs and PMN-MDSCs were significantly lower than in the untreated group when combinations of modified exosomes together with DC/mTMv were applied (Figures 5G,H). The obtained data also showed a considerably decreased percentage of resident macrophages in TEx MC38/shTGFβ1 + DC/TMv MC38/shTGFβ1 and TEx MC38/IL12shTGFβ1 + DC/TMv MC38/IL12shTGFβ1 groups (Figure 5J). We did not observe any changes in the percentage of TAM—the main subpopulation of macrophages in MC38 tumor nodules. However, the M1/M2 rate, which shows the influence of the applied therapy on macrophage polarization, indicates that treatment with combinations of mTEx and DCs stimulated with mTMv (especially with TEx MC38/IL12 + DC/TMv MC38/IL12 and TEx MC38/shTGFβ1 + DC/TMv MC38/shTGFβ1) caused the change of polarization of tumor-infiltrating macrophages (both TAM and Mf) toward M1 (Figure 5I). To confirm the influence of the applied immunotherapy on tumor-derived macrophage polarization we performed functional assays, which demonstrated the induction of the Th1 response, corroborating the shift of macrophage polarization toward M1 type. As presented in Figure 6A, statistically significant changes in the percentage of spleen-derived Tbet^+^IFN-γ^+^ Th lymphocytes, corresponding to the shift of the immune response toward Th1 type, were visible only in the group treated with TEx MC38/shTGFβ1 + DC/TMv MC38/shTGFβ1. In the remaining groups treated with mTEx and DC/mTMv the percentage of Th1 lymphocytes was also considerably higher than in the untreated control group. However, it was similar to values obtained for groups which received only DC/mTMv. Taking into account the decreased number of Treg cells among splenic CD4^+^ lymphocytes (Figure 6B), we also noted that treatment with TEx MC38/IL12 + DC/TMv MC38/IL12 or TEx MC38/shTGFβ1 + DC/TMv MC38/shTGFβ1 contributed to a significant increase in the proportion of Th1 to Treg cells (Figure 6C). The obtained data indicate that combined therapy with mTEx and DC/mTMv, especially application of TEx MC38/shTGFβ1 + DC/TMv MC38/shTGFβ1, is efficient in activation of a potent systemic Th1 response.

## Discussion

Scientific reports provide information confirming that cargo-loaded extracellular vesicles (EVs) have shown promising therapeutic effects in a variety of disease models, including cancer (11, 15, 31, 32). Recent research also demonstrated that tumor-derived exosomes, despite their high protumor activity, could become a very effective tool for delivery of chemotherapeutic drugs. Tang and co-workers, as well as Silva and co-workers, reported that chemotherapeutic drugs (methotrexate, cisplatin) and photosensitizers (m-THPC), respectively, can be effectively packed into tumor-derived EVs and used to inhibit tumor growth in murine cancer models (9, 33). It was also discovered that paclitaxel loaded into TEx showed 50 times higher cytotoxic activity than free paclitaxel and caused significant inhibition of lung metastasis growth (34). Besides drug delivery, tumor-derived EVs, due to their biocompatibility and directed migration, are also considered as prime carriers of other types of cargo, such as immunomodulating factors (in the form of non-coding RNA, mRNA, and protein).

The main objective of the study was to determine whether EVs released by tumor cells genetically engineered for overexpression of proinflammatory IL-12 and/or shRNA targeting suppressor TGF-β1 may lose protumor activity and acquire antitumor potential. According to a hypothesis, genetically induced overproduction of antitumor molecules could create a situation where tumor cells would produce protumor molecules at a reduced rate and start loading of the overexpressed molecules into EVs, thereby contributing toward raising the antitumor activity of the latter. Taking that into consideration, in the first stage of the study we confirmed the effectiveness of loading EVs with IL-12 and presence of TGF-β1 in the lumen of isolated particles. The results indicated that genetic modification of wild-type tumor cells is an effective method of loading the molecules of interest into extracellular vesicles secreted by the cells (both TEx and TMv). We observed that EVs released by MC38/IL12 and MC38/IL12shTGFβ1 contained IL-12 in the form of mRNA and active protein, while EVs isolated from MC38/shTGFβ1 or MC38/shIL12shTGFβ1 were characterized by significantly diminished level of TGF-β1 (both in the form of mRNA and protein). The effectiveness of genetic engineering of donor cells to modify the content of exosomes has also been confirmed by other research groups (13, 14, 35). Since, EVs are a source of tumor antigens, our further analyses focused on the influence of mTEx and mTMv as well as TEx and TMv obtained from wild-type MC38 cells on the activity of bone marrow-derived dendritic cells. As expected, modified EVs (both mTEx and mTMv) supported differentiation of DCs toward mature cells capable of presenting tumor antigens to naïve T lymphocytes. However, there were no significant differences between activity of DCs stimulated with EVs isolated from modified and wild-type MC38 cells. Additionally, we observed that DCs stimulated with TEx from wild-type MC38 cells and mTEx seemed to be better activators of antitumor activity of lymphoid cells than TMv MC38 and mTMv. It may be connected with different size of the applied particles. TEx with approximate size of 100 nm can be recognized and taken up by DCs as virus particles, whereas TMv with size >200 nm are recognized as bacteria. The different ways of processing EVs may result in differences in the stimulation efficacy of DCs, although TEx and TMv do not vary especially in a content. It can also be reflected in a diverse expression of MHC II on the surface of DCs stimulated with TEx or TMv. Furthermore, when we compared the effect of TEx MC38 stimulation with tumor lysate (TAg) stimulation—the most frequent method of tumor antigen delivery to DC-based vaccines—we noted that TEx MC38 were better stimulators than TAg. It was observable not only at the level of phenotypic changes occurring in DCs but also at the level of their functional activity. Similar results were demonstrated by Bu et al. (36). They found that T cells primed by DCs stimulated with TEx showed significantly higher cytotoxicity toward glioma cells than those stimulated with TAg. Our research also revealed the direct influence of modified EVs on the activity of spleen cells. We noted that splenocytes stimulated with mTEx or mTMv, especially EVs containing IL-12, produced high amounts of IFN-γ, while splenocytes stimulated with unmodified TEx or TMv produced IFN-γ at the same level as unstimulated cells.

The aim of our subsequent experiments was to confirm the antitumor potential of mTEx in *in vivo* conditions. Taking into consideration the high potential of TEx MC38 and mTEx to activate DCs, we decided to apply them in the form of sole treatment or in combination with DC-based vaccines. The obtained data showed that TEx from unmodified MC38 cells accelerated tumor growth, whereas mTEx, especially those deprived of TGF-β1, caused tumor growth inhibition. Although the combination of TEx MC38/shTGFβ1 with DC/TMv MC38/shTGFβ1 did not further improve the TEx MC38/shTGFβ1 therapeutic effect, we noted that application of TEx MC38/IL12shTGFβ1 with DC/TMv MC38/IL12TGFβ1 was able to induce over 70% tumor growth inhibition. By contrast, TEx MC38/IL-12 induced minor tumor growth inhibition at the level of 25%.

It also needs to be highlighted that only in groups of mice receiving a combination of mTEx and DCs/mTMv could we observe a statistically significant increase in the percentage of tumor-infiltrating CTLs and NK cells, which was accompanied by a reduction of suppressor MDSCs and Treg cells as well as favorable changes in the polarization of macrophages infiltrating tumor nodules. The obtained data confirm the important role of DC-based vaccines in regeneration of the immune response and induction of a systemic reaction. However, it also shows the necessity of supporting their action by other factors capable of reprogramming the hostile tumor microenvironment. Both selected cytokines play an important role in functioning of DCs. IL-12 induces differentiation of DCs and plays an essential role as the third signal during formation of a fully functional immunological synapse and activation of a Th1 type response (37, 38). On the other hand, TGF-β1 is a strong inhibitor of DCs. Moreover, it induces differentiation of DCs toward regulatory cells characterized by high protumor activity (39). Tumor-derived exosomes isolated from cells with overexpression of both IL-12 and shTGFβ1 can play a dual role in TME. Firstly, they can deliver IL-12, without the “negative cargo” in the form of suppressor TGF-β1, and they support differentiation of myeloid cells, effective presentation of tumor antigens by DCs and induction of a specific antitumor response. Secondly, siRNA against TGF-β1 constitutively produced in large amounts by modified tumor cells can also be loaded by the cells into EVs and may play a significant role in silencing of the TGF-β1 expression in target cells, thus hindering their protumor activity (35). Although both of mentioned factors (IL-12 and shRNA for TGF-β1) are very important for effective reactivation of immune response to fight cancer, our research revealed that the influence of TEx MC38/shTGFβ1 on the activation of specific antitumor response as well as reduction of immunosuppression in TME is significantly higher than the effect of treatment with TEx MC38/IL12. We suppose that the effect of the IL-12 delivered by TEx may be limited due to high activity of suppressor cells such as MDSCs or Treg in the TME. By contrast, TEx MC38/shTGFβ1 deliver tumor antigens without the “negative cargo” in the form of TGF-β1, thereby the response of immune cells for tumor antigens is more efficient. Additionally, we suppose that TEx MC38/shTGFβ1 may support the reactivation of antitumor response by delivery of siRNA for TGF-β1, which reduce the immunosuppression inside a tumor. In our previous study we used lentivectors encoding shTGFβ1 to reduce the concentration of the cytokine in TME and enhance the antitumor activity of DC-based immunotherapy. Although the final effect of the treatment was spectacular (97% TGI), we noted high immunogenicity of the applied lentivectors (20). Compared to lentivectors, TEx seems to be significantly better delivery vectors due to their biocompatibility, targeted migration, and versatility. Moreover, it seems probable that modified exosomes, in contrast to other delivery vectors, can have a the wider spectrum of action. Certainly, peritumorally injected exosomes carrying immunomodulatory factors such as IL-12 and shRNA for TGF-β1 can take part in the reprogramming of suppressive TME and supporting the antitumor activity of peritumorally injected DC-based vaccines as well as reactivating endogenous immune cells arrested in a tumor nodule. In addition, due to their high stability and biocompatibility, modified tumor-derived exosomes could also migrate toward draining lymph nodes, where they could directly support dendritic cells in effective activation of naïve T cells, or to presumable metastatic sites specific for a tumor, where they could prevent formation of a premetastatic niche. However, the hypothesis should be confirmed in further research.

Taken together, the presented results indicate that tumor-derived exosomes secreted by cells engineered for overproduction of IL-12 and shRNA for TGF-β1 are able to induce tumor growth inhibition as opposed to TEx from unmodified MC38 cells. Moreover, their application (especially those deprived of TGF-β1) with DC-based vaccines allowed for regeneration of antitumor immunity and induction of systemic the Th1 response responsible for the sustained effect of the therapy. Nevertheless, further research is required to provide better knowledge of the changes occurring in mTEx following genetic modifications and to determine possible side effects of their application.

## Ethics Statement

This study was carried out in accordance with EU Directive 2010/63/EU for animal experiments and were approved by the 1st Local Ethics Committee for Experiments with the Use of Laboratory Animals, Wroclaw, Poland (authorization number 33/2018).

## Author Contributions

JR: substantial contribution to the conception and design of the research, planning, and performing experiments, data analysis, interpretation, and drafting the manuscript; NA: planning and performing *in vitro* and *ex vivo* experiments and data analysis; KW, AS, and JM: planning and performing *ex vivo* experiments; MM: performing western blotting analyses; BS-O: EV visualization by TEM—optimization of the method and sample preparation and analysis; EP-P: planning and performing *in vivo* experiments. All authors reviewed the manuscript and approved its final version.

### Conflict of Interest Statement

The authors declare that the research was conducted in the absence of any commercial or financial relationships that could be construed as a potential conflict of interest.

## Supplementary Material

Supplementary Figure 1 Evaluation of the number of TEx and TMv secreted by the wild-type and genetically modified MC38 cells per isolation performed by flow cytometry using Absolute Counting Beads. Bar graphs present the mean ± SD calculated for three repeats. The differences between the groups were estimated using the nonparametric Kruskal-Wallis test followed by Dunn's multiple comparison test (^*^*p* < 0.05, ^***^*p* < 0.001).

Supplementary Figure 2 Flow cytometry analysis of TEx and TMv isolated from wild-type or genetically modified MC38 cell lines. Histograms represent the expression of CD63, CD9, CD81 on the surface of TEx and TMv. Bar graphs present the mean ± SD calculated for three repeats. The differences between the groups were estimated using the nonparametric Kruskal-Wallis test followed by Dunn's multiple comparison test (^*^*p* < 0.05).

Supplementary Figure 3 Concentration of IFN-γ in supernatants from spleen cells stimulated with TEx or TMv, isolated from wild-type or genetically modified MC38 cell lines. Bar graphs present the mean ± SD calculated for three repeats. The differences between the groups were estimated using the nonparametric Kruskal-Wallis test followed by Dunn's multiple comparison test (^*^*p* < 0.05, ^****^*p* < 0.0001).

## Abbreviations

EVs: extracellular vesicles
mTEx: tumor-derived exosomes secreted by genetically modified cells
mTMv: tumor-derived microvesicles secreted by genetically modified cells
DCs: dendritic cells
MC38: murine colon carcinoma
MDSCs: myeloid-derived suppressor cells.
