# Supplementary figures and images for: Antitumor Potential of Extracellular Vesicles Released by Genetically Modified Murine Colon Carcinoma Cells With Overexpression of Interleukin-12 and shRNA for TGF-β1
Source: Front Immunol. 2019 Feb 13;10:211. doi: 10.3389/fimmu.2019.00211 (PMC6381037; doi:10.3389/fimmu.2019.00211)

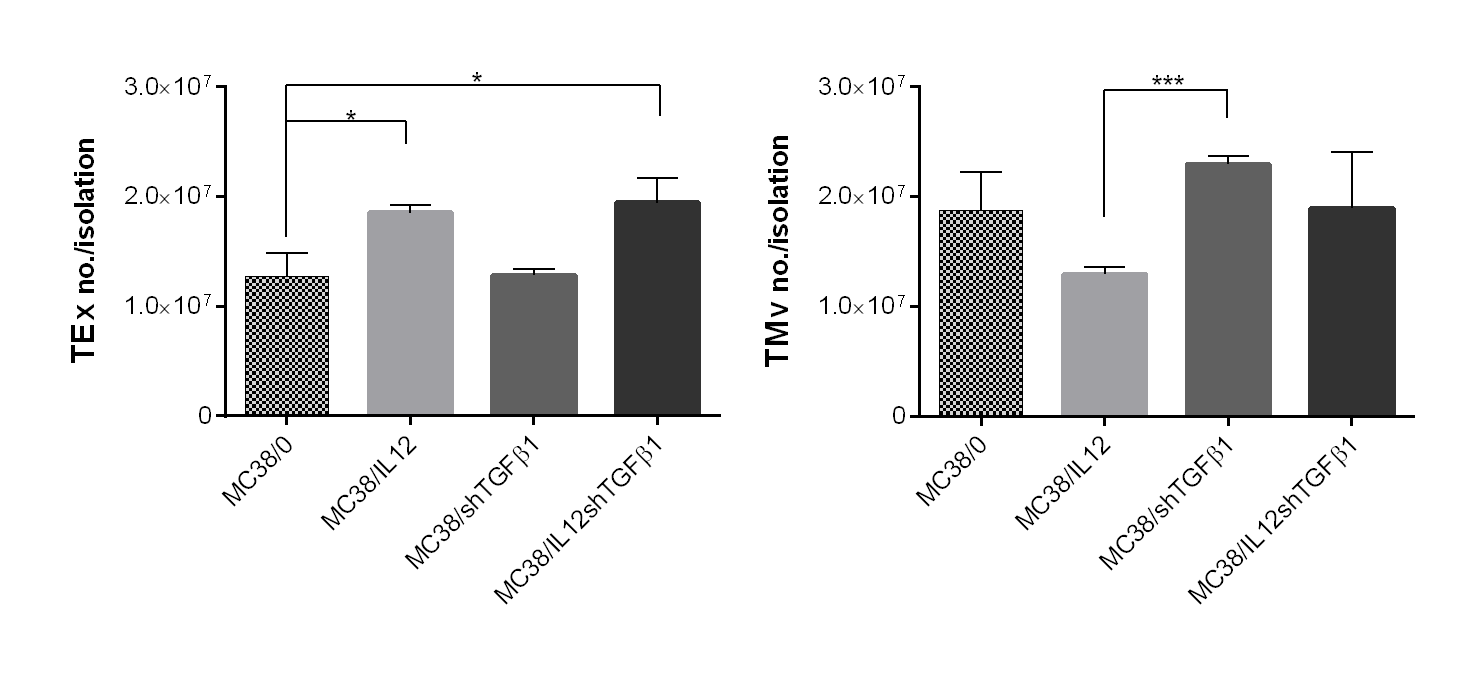

Supplement: Supplementary Figure 1 — Evaluation of the number of TEx and TMv secreted by the wild-type and genetically modified MC38 cells per isolation performed by flow cytometry using Absolute Counting Beads. Bar graphs present the mean ± SD calculated for three repeats. The differences between the groups were estimated using the nonparametric Kruskal-Wallis test followed by Dunn's multiple comparison test (*p < 0.05, ***p < 0.001). [file Image_1.TIF]

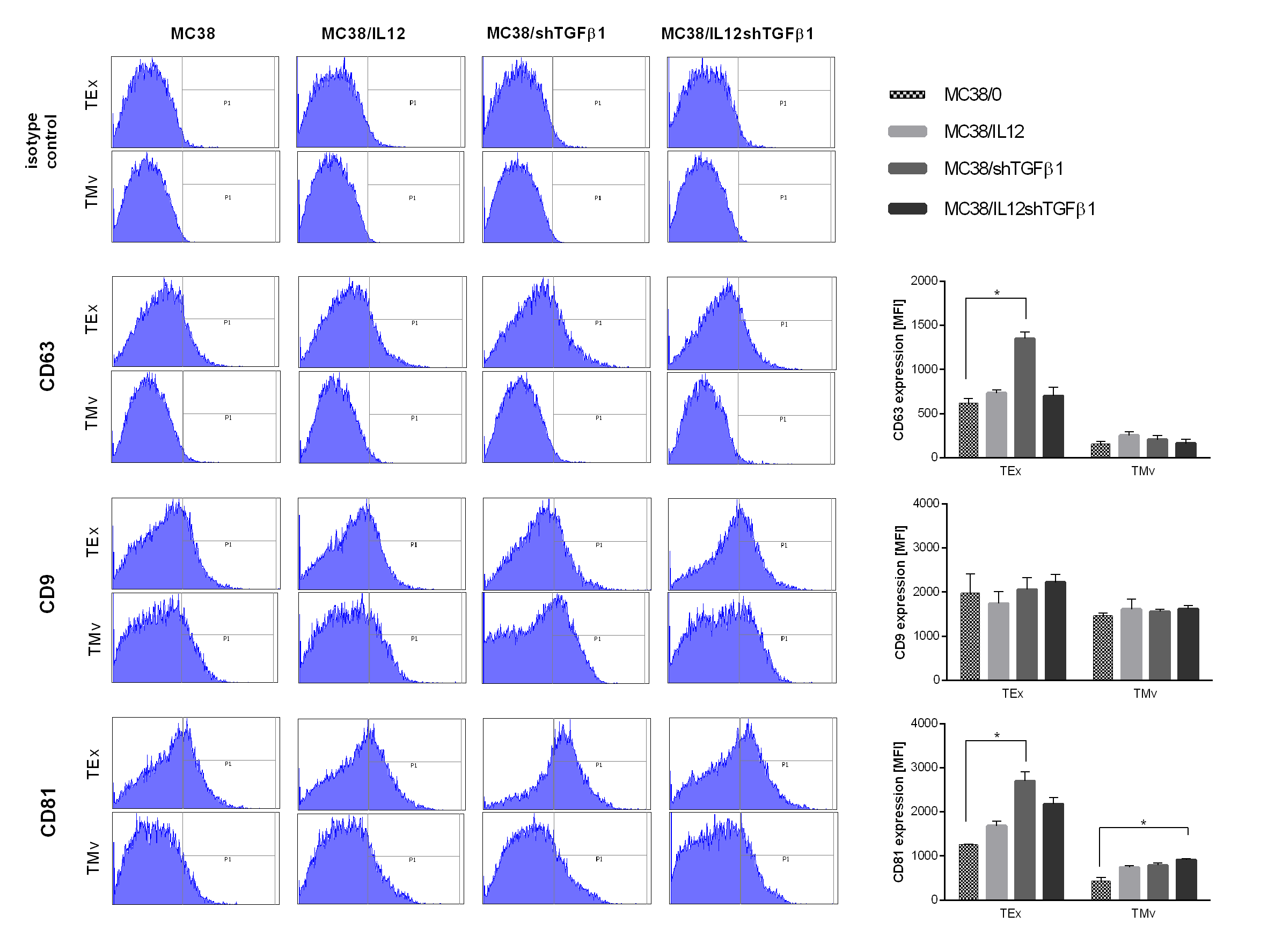

Supplement: Supplementary Figure 2 — Flow cytometry analysis of TEx and TMv isolated from wild-type or genetically modified MC38 cell lines. Histograms represent the expression of CD63, CD9, CD81 on the surface of TEx and TMv. Bar graphs present the mean ± SD calculated for three repeats. The differences between the groups were estimated using the nonparametric Kruskal-Wallis test followed by Dunn's multiple comparison test (*p < 0.05). [file Image_2.TIF]

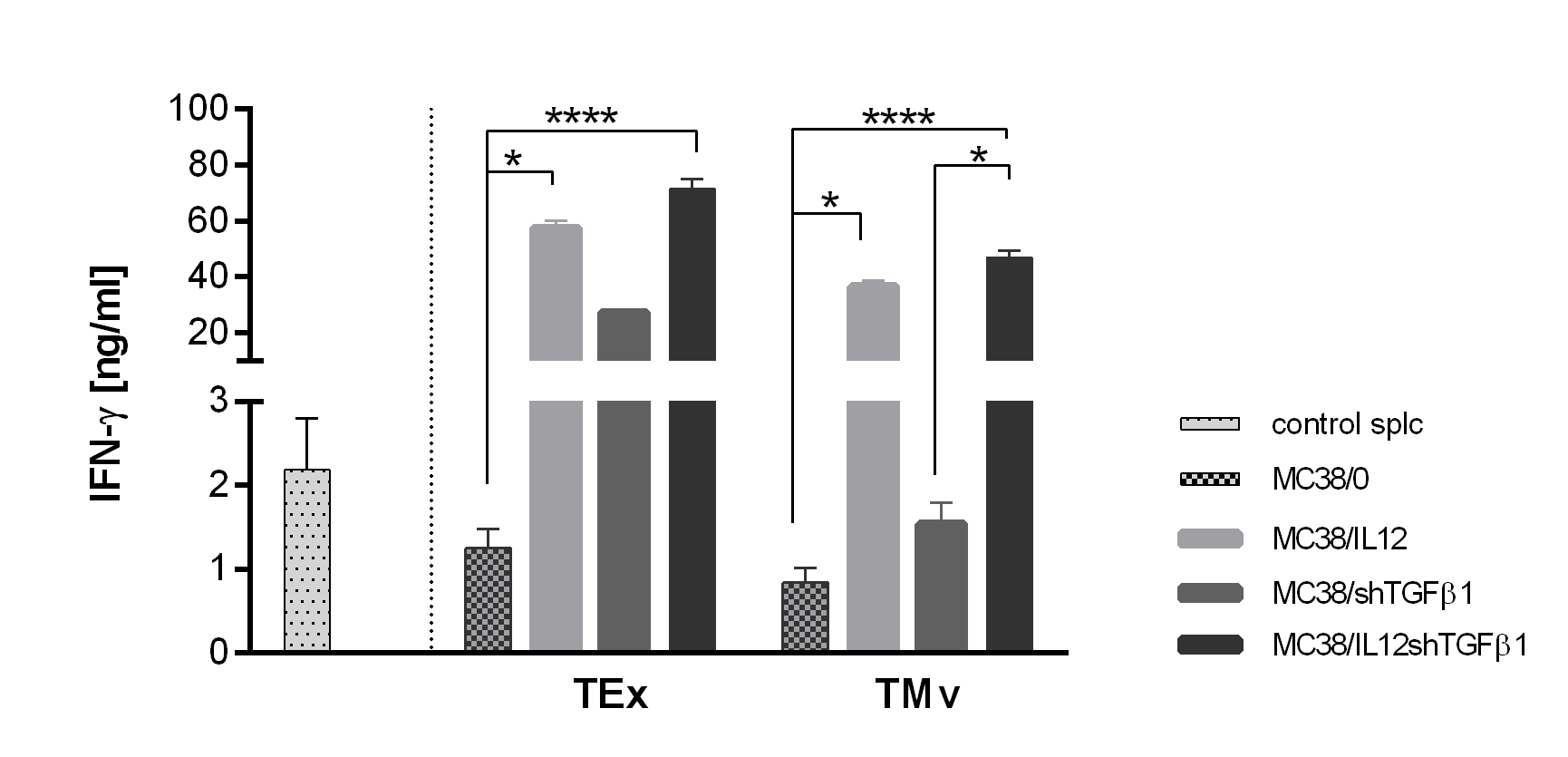

Supplement: Supplementary Figure 3 — Concentration of IFN-γ in supernatants from spleen cells stimulated with TEx or TMv, isolated from wild-type or genetically modified MC38 cell lines. Bar graphs present the mean ± SD calculated for three repeats. The differences between the groups were estimated using the nonparametric Kruskal-Wallis test followed by Dunn's multiple comparison test (*p < 0.05, ****p < 0.0001). [file Image_3.TIF]
